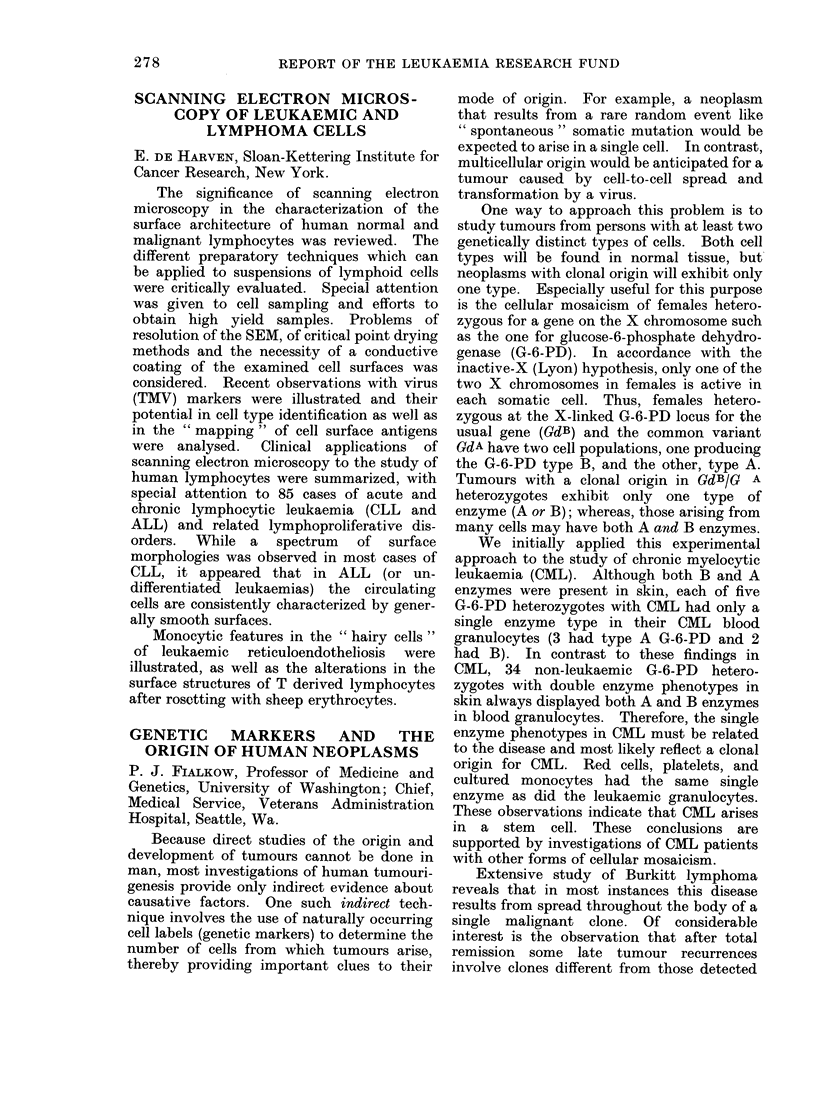# Proceedings: Scanning electron microscopy of leukaemic and lymphoma cells.

**DOI:** 10.1038/bjc.1975.214

**Published:** 1975-08

**Authors:** E. de Harven


### SCANNING ELECTRON MICROSCOPY OF LEUKAEMIC AND LYMPHOMA CELLS

E. de Harven, Sloan-Kettering Institute for Cancer Research, New York.

The significance of scanning electron microscopy in the characterization of the surface architecture of human normal and malignant lymphocytes was reviewed. The different preparatory techniques which can be applied to suspensions of lymphoid cells were critically evaluated. Special attention was given to cell sampling and efforts to obtain high yield samples. Problems of resolution of the SEM, of critical point drying methods and the necessity of a conductive coating of the examined cell surfaces was considered. Recent observations with virus (TMV) markers were illustrated and their potential in cell type identification as well as in the " mapping " of cell surface antigens were analysed. Clinical applications of scanning electron microscopy to the study of human lymphocytes were summarized, with special attention to 85 cases of acute and chronic lymphocytic leukaemia (CLL and ALL) and related lymphoproliferative disorders. While a spectrum of surface morphologies was observed in most cases of CLL, it appeared that in ALL (or undifferentiated leukaemias) the circulating cells are consistently characterized by generally smooth surfaces.

Monocytic features in the " hairy cells " of leukaemic reticuloendotheliosis were illustrated, as well as the alterations in the surface structures of T derived lymphocytes after rosetting with sheep erythrocytes.